# NY-ESO-1 Cancer Testis Antigen Demonstrates High Immunogenicity in Triple Negative Breast Cancer

**DOI:** 10.1371/journal.pone.0038783

**Published:** 2012-06-28

**Authors:** Foluso O. Ademuyiwa, Wiam Bshara, Kristopher Attwood, Carl Morrison, Stephen B. Edge, Christine B. Ambrosone, Tracey L. O’Connor, Ellis G. Levine, Anthony Miliotto, Erika Ritter, Gerd Ritter, Sacha Gnjatic, Kunle Odunsi

**Affiliations:** 1 Department of Medicine, Roswell Park Cancer Institute, Buffalo, New York, United States of America; 2 Department of Pathology, Roswell Park Cancer Institute, Buffalo, New York, United States of America; 3 Department of Biostatistics, Roswell Park Cancer Institute, Buffalo, New York, United States of America; 4 Department of Surgical Oncology, Roswell Park Cancer Institute, Buffalo, New York, United States of America; 5 Department of Cancer Prevention and Control, Roswell Park Cancer Institute, Buffalo, New York, United States of America; 6 Department of Gynecologic Oncology, Roswell Park Cancer Institute, Buffalo, New York, United States of America; 7 Ludwig Institute for Cancer Research Ltd. New York Branch of Human Cancer Immunology, Memorial Sloan Kettering Cancer Center, New York, New York, United States of America; Baylor College of Medicine, United States of America

## Abstract

**Purpose:**

NY-ESO-1 cancer testis (CT) antigen is an attractive candidate for immunotherapy as a result of its high immunogenicity. The aim of this study was to explore the potential for NY-ESO-1 antigen directed immunotherapy in triple negative breast cancer (TNBC) by determining the frequency of expression by immunohistochemistry (IHC) and the degree of inherent immunogenicity to NY-ESO-1.

**Experimental Design:**

168 TNBC and 47 ER+/HER2- primary breast cancer specimens were used to determine NY-ESO-1 frequency by IHC. As previous studies have shown that patients with a robust innate humoral immune response to CT antigens are more likely to develop CD8 T-cell responses to NY-ESO-1 peptides, we evaluated the degree to which patients with NY-ESO-1 expression had inherent immunogenicity by measuring antibodies. The relationship between NY-ESO-1 expression and CD8+ T lymphocytes was also examined.

**Results:**

The frequency of NY-ESO-1 expression in the TNBC cohort was 16% versus 2% in ER+/HER2- patients. A higher NY-ESO-1 score was associated with a younger age at diagnosis in the TNBC patients with NY-ESO-1 expression (p = 0.026). No differences in OS (p = 0.278) or PFS (p = 0.238) by NY-ESO-1 expression status were detected. Antibody responses to NY-ESO-1 were found in 73% of TNBC patients whose tumors were NY-ESO-1 positive. NY-ESO-1 positive patients had higher CD8 counts than negative patients (p = 0.018).

**Conclusion:**

NY-ESO-1 is expressed in a substantial subset of TNBC patients and leads to a high humoral immune response in a large proportion of these individuals. Given these observations, patients with TNBC may benefit from targeted therapies directed against NY-ESO-1.

## Introduction

Contemporary management of breast cancer with early detection, newer local control techniques, improved chemotherapy regimens, and targeted treatments has resulted in immense gains in survival in individuals with breast cancer.[1] Unfortunately, the triple negative breast cancers (TNBC) which are a subset of breast cancers clinically defined by the absence of the estrogen receptor (ER), progesterone receptor (PR), and Her 2 over expression, lack a therapeutic target and have a poor prognosis. Compared with non-TNBC, these lesions generally occur in younger women, are of a higher grade, have a higher propensity to metastasize to distant visceral organs, and have a worse outcome with a high rate of recurrences after adjuvant treatments.[2] Thus, there is a dire need to develop tumor-specific targets in an attempt to improve the outcome for patients with TNBC. An attractive approach to reduce the rate of recurrences in these individuals is use of immunotherapeutic strategies which will be most efficient in the state of minimal residual disease in individuals who have completed standard surgery and adjuvant treatments. A pre-requisite for the development of immune therapies is the identification of immunogenic target cancer antigens.

Cancer testis (CT) antigens are encoded by a unique set of genes that are predominantly expressed in human germ line cells and have minimal to no expression in somatic adult tissue. They become abnormally activated in a variety of malignancies including ovary, bladder, synovial sarcoma, lung, melanoma, and breast cancer with over one hundred and fifty CT antigens described.[3], [4], [5], [6], [7], [8] The physiological function or prognostic implication of most of the CT antigens remains unknown. NY-ESO-1 is one of the more prominent CT antigens and is located on the X-chromosome. It is found in a variety of tumors with different histologic origins but not in normal tissues other than the testis. NY-ESO-1 is believed to be one of the most immunogenic CT antigens, inducing spontaneous humoral immunity in a subset of patients whose tumors express this antigen.[9], [10], [11] As a result of this property, NY-ESO-1 is an attractive candidate for immunotherapy. Several early-phase clinical trials employing NY-ESO-1 vaccines have demonstrated the ability of the vaccine to induce T-cell and antibody mediated immunity.[12], [13], [14], [15], [16].

In this study, we analyzed the frequency of NY-ESO-1 expression in a large cohort of TNBC patient samples using immunohistochemistry (IHC) and also examined NY-ESO-1 expression in relation to patient clinicopathologic characteristics and degree of tumor infiltration by CD8+ T lymphocytes (TILs). Because patients with robust humoral immunity to CT antigens are more likely to have concomitant CD8 T-cell responses to NY-ESO-1,[17] we evaluated the degree to which patients whose tumors expressed NY-ESO-1 had inherent immunogenicity by measuring humoral immunity to NY-ESO-1 and other CT antigens. To our knowledge, this is the most comprehensive study of CT antigens in TNBC.

**Table 1:** Patient characteristics.

		TNBC		ER+/HER2-		p value
		N	%	N	%	
All samples		168	100	47	100	
Age at diagnosis	Mean (SD)	52.9 (13)		58.4 (15.4)		
Race	White	117	70	45	96	<.001
	Black	47	28	2	4	
Grade	1	3	2	6	13	<.001
	2–3	160	95	29	62	
Histology	Ductal	151	90	29	62	<.001
	Lobular	7	4	9	19	
	Other	10	6	9	19	
Stage^1^	1	1	1	-	-	<.001
	2	109	65	-	-	
	3	56	33	43	91	
	4	1	1	4	9	
Tumor size^1^	Mean (SD)	3.3 (2.3)		4.0 (2.5)		.025
LVI^2^	No	69	49	9	43	.645
	Yes	71	51	12	57	

1 These are not accurate reflections of the differences between TNBC and ER+/HER2- due to the sampling method.

2 LVI is lymphovascular invasion.

## Materials and Methods

### Patients and Specimens

A total of 215 formalin-fixed paraffin embedded breast cancer specimens were obtained from Roswell Park Cancer Institute (RPCI) pathology resource network from patients who had been treated between 1996 and 2010. For a subset of patients whose tumors were found to express NY-ESO-1 by IHC, serum samples were retrieved from the RPCI Data Bank and BioRepository (DBBR). The DBBR, as previously described[18] is a comprehensive data and sample bank containing high quality biospecimens obtained prior to surgery and treatment from patients who provided informed consent, as well as associated clinical and epidemiologic data. Medical records were retrospectively reviewed on all patients for details of the clinicopathologic characteristics of the diagnosed cancer, including information on clinical outcome. The duration of overall survival (OS) was defined as the time period between diagnosis and death, while progression-free survival (PFS) was the time between diagnosis and evidence of clinical progression or death. Data were censored at time of last follow-up for those patients without an event.

### Ethics Statement

The study was conducted under approval from the RPCI Institutional Review Board.

**Figure 1:**
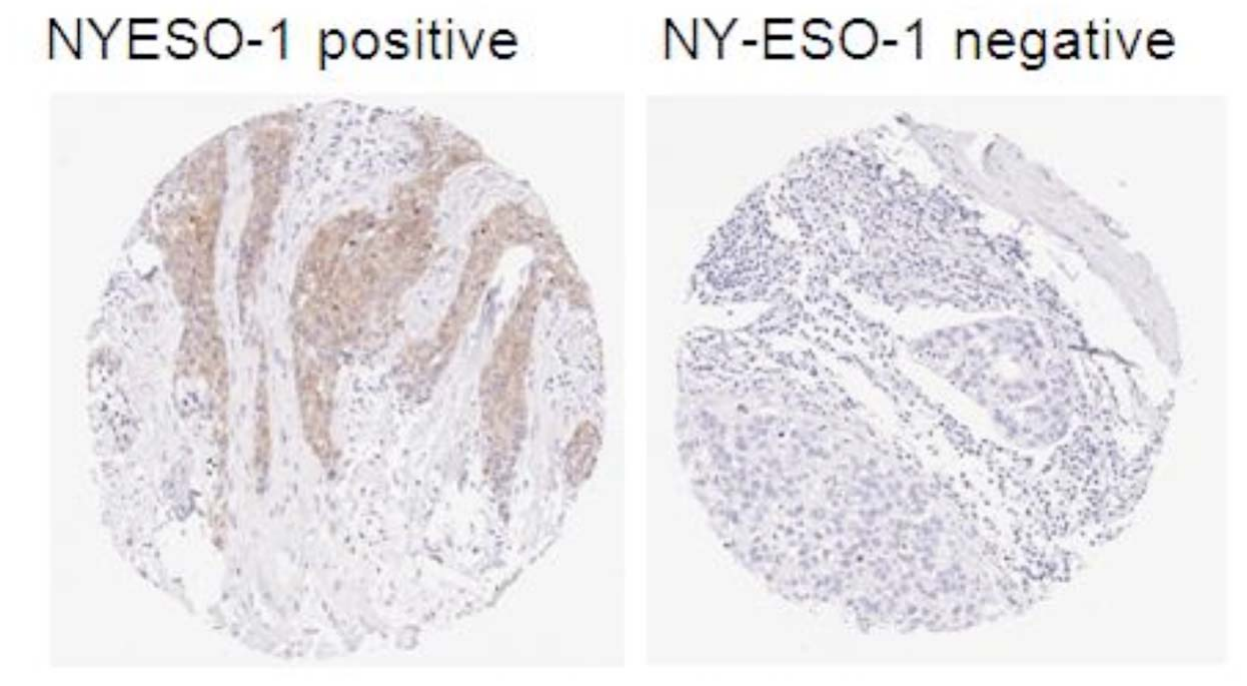
NY-ESO-1 expression by IHC compared with a negative IgG control. Photomicrograph of NY-ESO-1 expression by immunohistochemistry side by side compared with a negative IgG control.

**Table 2:** NY-ESO-1 expression frequency by IHC.

Expression	TNBC		ER+/HER2-	
	N (168)	%	N (47)	%
Intensity				
3+	11	7	0	-
2+	10	6	1	2
1+	6	3	0	-
**Total positive**	**27**	**16**	**1**	**2**
Total negative	141	84	46	98
Percent staining				
<10%	13	8	1	2
10–50%	5	3	0	-
>50%	9	5	0	-
**Total positive**	**27**	**16**	**1**	**2**
Total negative	141	84	46	98

### IHC

The methods for determining NY-ESO-1 expression from formalin-fixed paraffin sections have been previously described.[19], [20] In brief, paraffin sections were cut at 5 µm, placed on charged slides and dried in a 60°C oven for 1 hour. Room temperature slides were then deparaffinized in xylene and rehydrated using graded alcohols. Endogenous peroxidase was then quenched with aqueous hydrogen peroxidize and washed with phosphate buffered saline tween (PBS/T). Antigen retrieval was then performed in the microwave in Target Retrieval Solution (Dako), with a cool down period, followed by a PBS/T wash. Slides were then loaded on the DAKO autostainer and the following program was run: Casein 0.03% (in PBS/T) was used to block for 30 minutes, blown off, and the primary antibody NY-ESO-1 (Zymed/Invitrogen mouse monoclonal) applied at 5 µg/ml to slides for one hour. A PBS/T wash was followed by mouse Envision+ Polymer (Dako). PBS/T was then used as a wash and the chromagen DAB+ (Dako) applied for 10 minutes. The slides were then counterstained with hematoxylin, dehydrated, cleared, and coverslipped. Antigen expression was scored by a trained pathologist (WB) and was categorized based on the percentage staining (0%, <10%, 10–50%, >50%) and intensity (0, 1, 2, 3) of the tumor cell population. Methods used to determine CD8 testing by IHC have been described previously.[12] Each entire tumor section was evaluated for tumor infiltrating lymphocytes (TILs) by using X20 objective lens, and 10 independent areas with the most abundant TILs selected, digitally photographed at a size of 0.0625 mm^2^, and counted manually. The count was performed 2 times for each photograph by the same pathologist without knowledge of the earlier result. The average TIL count for each patient was used for statistical analysis. The average CD8 count per 1 mm^2^ was computed.

**Figure 2:**
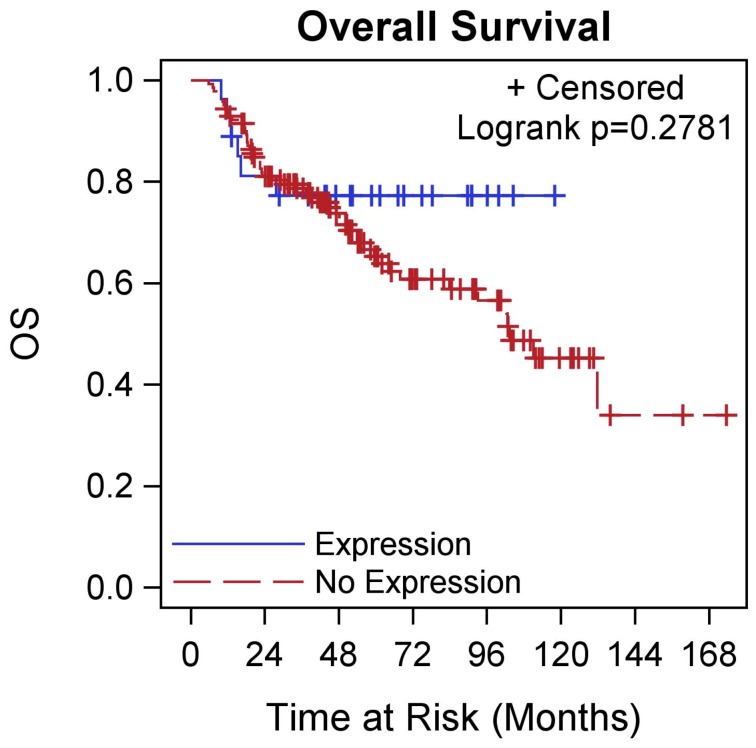
OS by NY-ESO-1 expression in TNBC. Kaplan-Meier curve showing OS differences in patients with TNBC stratified by NY-ESO-1 expression.

**Figure 3:**
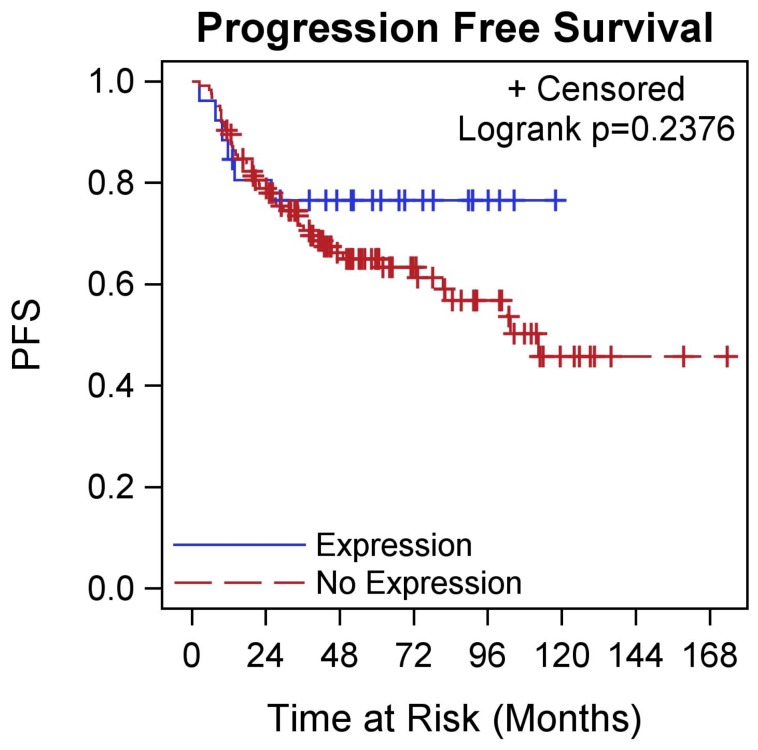
PFS by NY-ESO-1 expression in TNBC. Kaplan-Meier curve showing PFS differences in patients with TNBC stratified by NY-ESO-1 expression.

### Detection of CT Antibodies by ELISA

Human sera samples which had been stored in liquid nitrogen vapor were obtained from the RPCI DBBR for a subset of patients. Antibodies to NY-ESO-1 and other CT antigens were detected by ELISA as described previously.[21].

### CT Antigen Gene Expression and Promoter DNA Hypomethylation

MCF-7 breast cancer cell line (originally procured from American Type Culture Collection, Manassas, VA) was a gift from Dr. Gokul Das (Department of Pharmacology and Therapeutics, RPCI). The cell line was grown in the recommended media under standard conditions. Cells were treated with either Decitabine (DAC, 5-aza-2′-deoxycytidine, Sigma-Aldrich Corp, St. Louis, MO) at 0.1, 0.5, or 1 µM or Vidaza (5-azacytidine, Sigma-Aldrich Corp, St. Louis, MO) at 1 or 2 µM. Cells were treated twice at 0 and 48 hours, and RNA and DNA were extracted at 72 hours for analyses. Total RNA was extracted using Trizol (Invitrogen, Carlsbad, CA) and cDNA synthesis was carried out with iscript cDNA synthesis kit (Biorad, Hercules, CA). NY-ESO-1 was amplified using the following primers: F 5′GGCTGAATGGATGCTGCAGA3′and R 5′ CGGACACAGTGAA CTCCTTC-3′ for 35 cycles at 60°C. GAPDH was amplified as a control for equal cDNA input. With regards to sodium bisulfite DNA pyrosequencing, briefly, DNA was isolated using Puregene core kit A (Qiagen, Valencia, CA). EZ DNA methylation kit (Zymo Research Corp, Irvine, CA) was used to bisulfite convert 1 ug of DNA according to the manufacturer’s recommendations. Quantitative pyrosequencing of NY-ESO-1 promoter was performed on a PSQ 96 ID pyrosequencer (Qiagen, Valencia, CA). The primers used for amplification and sequencing of NY-ESO-1 and the conditions used were as previously described.[22].

**Table 3:** Characteristics of NY-ESO-1 positive TNBC patients with NY-ESO-1 antibodies.

Patient ID	Age atdiagnosis	Stage	Grade	Recurrence	Status at lastfollow up	Disease free interval in weeks
1	60	lllA T2 N2 M0	3	NONE, DISEASE FREE	ALIVE	351
2	41	IIA T1 N1 M0	3	NONE, DISEASE FREE	ALIVE	279
3	49	lllC T1 N3 M0	3	NONE, DISEASE FREE	ALIVE	292
4	56	IIA T2 N0 M0	3	NONE, DISEASE FREE	ALIVE	247
5	50	IIA T2 N0 M0	3	NONE, DISEASE FREE	ALIVE	165
6	40	IIB T2 N1 M0	3	NONE, DISEASE FREE	ALIVE	241
7	56	IIA T2 N0 M0	3	NONE, DISEASE FREE	DEAD (pancreas cancer)	50
8	39	IIB T2 N1 M0	3	NONE, DISEASE FREE	ALIVE	301

**Table 4:** Characteristics of NY-ESO-1 positive TNBC patients without NY-ESO-1 antibodies.

Patient ID	Age at diagnosis	Stage	Grade	Recurrence	Status at last follow up	Disease free interval in weeks
9	44	IIB T2 N0 M0	3	NONE, DISEASE FREE	ALIVE	217
10	45	IIA T2 N0 M0	2	NONE, DISEASE FREE	ALIVE	552
11	73	IIIA T2 N2 M0	3	NONE, DISEASE FREE	ALIVE	83

### Statistical Analyses

The categorical variables were summarized using frequencies and relative frequencies. Fisher’s exact test used to study the association between categorical variables and TNBC status. Numerical variables were summarized using means and standard deviations, with the Wilcoxon exact test used compare numerical variables between TNBC groups. For those patients with NY-ESO-1 expression, a score (1–9) was calculated as intensity (1 =  <10%, 2 = 10%–50% and 3 =  >50%) times expression (1 = 1+, 2 = 2+ and 3 = 3+). The association between this NY-ESO-1 score and patient demographics was assessed using univariate regression models, with the NY-ESO-1 score as the response and the demographic variables as predictors. Diagnostic plots were used to assess all model assumptions. The Kruskal Wallis exact test was used to compare the CD8 counts between the minimum, moderate, and brisk infiltrate groups for both the NYESO-1 positive and negative patients. The Wilcoxon exact test was used to compare the CD8 counts between the NYESO-1 positive and negative patients. The overall and progression free survival times were summarized using Kaplan-Meier methodology, with the Log-rank test used to compare the survival distributions between patient groups. All analysis was conducted in SAS v9.2 (Cary, NC) with a nominal significance level of 0.05.

**Figure 4:**
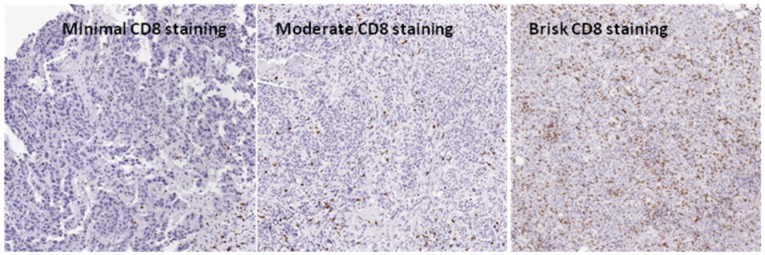
Extent of CD8 infiltration in triple negative tumors. This figure demonstrates the extent of CD8 infiltration in triple negative breast specimens.

**Figure 5:**
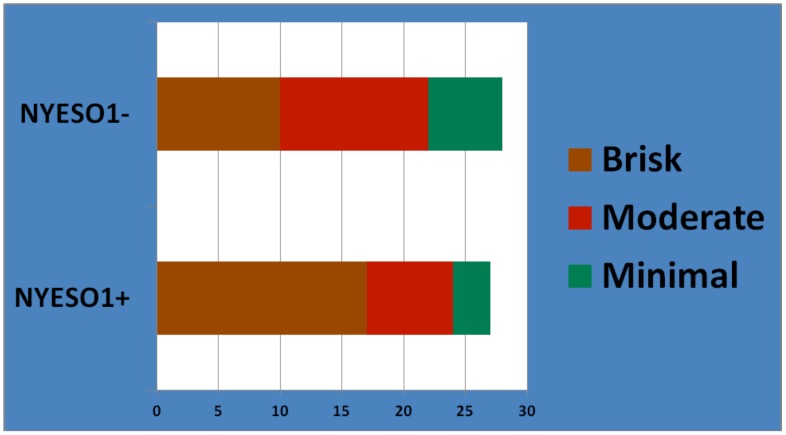
The relationship between NY-ESO-1 expression and degree of CD8 tumor infiltrating lymphocytes in TNBC. This figure shows the relationship between NY-ESO-1 expression and degree of CD8 tumor infiltrating lymphocytes in TNBC.

## Results

### Study Population

Two hundred and fifteen (168 TNBC and 47 ER+/HER2-) primary breast cancer patients’ specimens were used to determine the frequency of NY-ESO-1 CT antigen by IHC. ER+/HER2- tumors were used as a comparison to avoid the potential for confounding by HER+ status. We initially planned to select patients with more advanced stage and/or higher grade tumors where one would expect NY-ESO-1 to be positive based on the literature. However, in order to achieve the required sample size of TNBC patients, we had to include a large number of those cases with earlier stage disease. The ER+/HER2- cases were left as more advanced stage due to the availability of sufficient patient samples with this subtype. Table 1 shows the patient characteristics of the entire cohort. As expected, TNBC patients were more likely to be younger (p = 0.030), black (p<0.001), have ductal histology (p<0.001), and higher grade tumors (p<0.001) than patients with ER+ breast cancer despite the higher proportion of advanced stages in the ER+ patients due to the sampling method.

**Figure 6:**
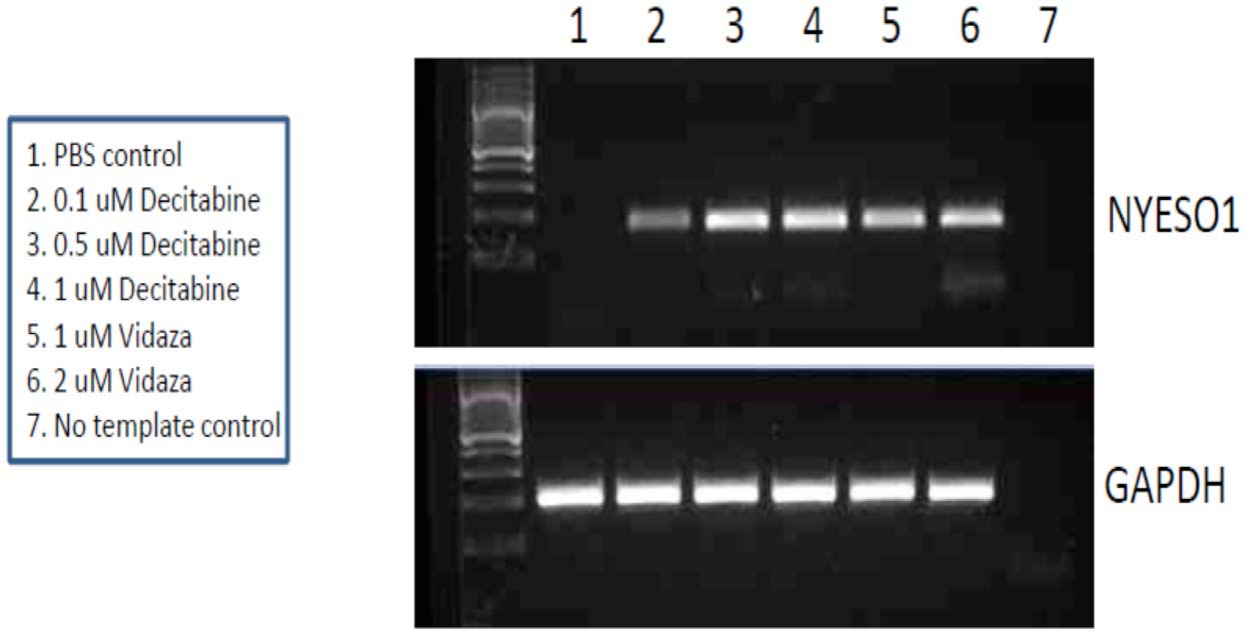
Induction of NY-ESO-1 by decitabine and 5-azacytidine. Photomicrograph showing the ability of decitabine and 5-azacytidine to induce of NY-ESO-1 *in vitro* in MCF-7 cells.

**Figure 7:**
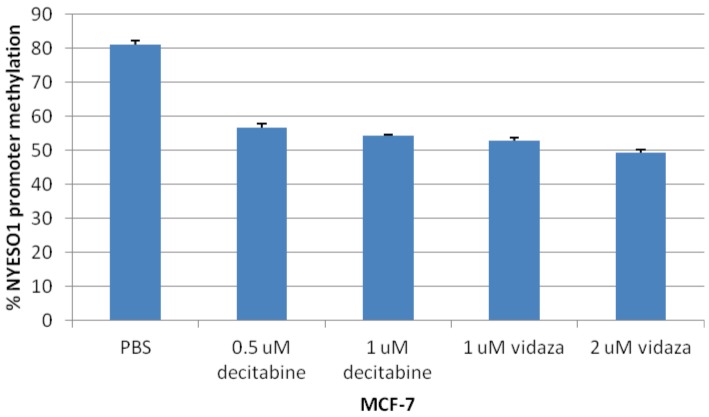
NY-ESO-1 promoter methylation was reduced by both decitabine and 5-azacytidine in MCF-7 cells. Graph showing that NY-ESO-1 promoter methylation was reduced by both decitabine and 5-azacytidine in MCF-7 cells.

### NY-ESO-1 Expression

To avoid the problem of tumor heterogeneity, whole sections were used to determine the frequency of NY-ESO-1 expression by IHC. Figure 1 shows a positively staining tumor in comparison with a negative control. In the entire breast cancer cohort, the frequency of NY-ESO-1 expression was 13%. In TNBC it was 16%, and just 2% in ER+ (Table 2). Most of the tumors expressing NY-ESO-1 positivity were either 2 or 3+ in intensity, but <10% in percentage staining. We also calculated a score of 0–9 which was a combination of the intensity and percent staining and found a significant association between intensity and percent in those who expressed NY-ESO-1.

In the 27 TNBC patients with NY-ESO-1 expression, we found that a higher NY-ESO-1 score was associated with a younger age at diagnosis (p = 0.026). There was no significant relationship between presence of lymphovascular space invasion, histology, stage, grade, tumor size, number of involved lymph nodes, or race. In the entire TNBC cohort, there were no differences in OS (p = 0.278) or PFS (p = 0.238) by NY-ESO-1 expression status (Figures 2 & 3). Only 6 deaths (all within 2 years) occurred out of 27 TNBC patients with expression and 51 deaths out of 141 TNBC patients whose tumors did not express NY-ESO-1. Patients had a similar 2 year OS rate of 77% (95% CI: 56–89%) vs 80% (95% CI: 73–86%). Similar results were found for PFS.

### Analysis of Immune Response to NY-ESO-1 CT Antigen in TNBC

Eleven of 27 NY-ESO-1 expressing TNBC patients had pre-therapy serum samples available to assess for antibody responses. Antibodies against NY-ESO-1, LAGE-1, several other CT antigens (MAGE-A1, MAGE-A3, p53, DHFR, MAGE-A4, MAGE-A10, SSX-1, SSX-2, SSX-4, SOX2, CT7, CT10, CT45, CT46, CT47, XAGE-1, PLAC1 MELAN-A, GAGE-2/7, CT55, CT63, TRAG3/CT24, ERG, CT39, CT14, CT57), Melan-A, and UBQLN2 were investigated. Antibody responses to NY-ESO-1 were found in 8 of 11 (72.7%) TNBC patients whose tumors were NY-ESO-1 positive by IHC. A similarly high proportion, (7 of 11; 64%) demonstrated spontaneous antibody response to LAGE-1, which has a 94% homology to NY-ESO-1.[23] Tables 3 and 4 show the characteristics of TNBC patients with and without an immune response to NY-ESO-1 CT antigen. The mean disease free interval was similar between the patients whose sera had NY-ESO-1 antibodies versus the group of patients that did not have innate antibody responses (240 versus 284 weeks). The only other antigen that had a substantial proportion of antibodies detected was p53 CT antigen; 4 of 11 (36.4%) patients. Antibody responses to the other antigens were infrequent ranging from 0 to 18%.

### NY-ESO-1 Expression and CD8 Tumor Infiltrating Lymphocytes (TILs)

To test whether NY-ESO-1 expression predicts infiltration by CD8+ TILs, we examined the relationship between NY-ESO-1 expression and CD8+ TILs. The CD8 count was calculated by dividing of the number of CD8 positive cells by the area of analysis. A minimal, moderate, or brisk CD8 infiltrate was characterized as 0–5, 6–50, or >50 cells/high power field respectively.[9] Figure 4 demonstrates the extent of CD8 infiltration in triple negative breast specimens. Among the 27 TNBC patients whose tumors were NY-ESO-1 positive by IHC, the median CD8 count was 358.5cells/mm^2^ (range 0.9 to 1601.1cells/mm^2^). Seventeen of these 27 patients (63%) had a brisk CD8 infiltrate, while a moderate or minimal infiltrate was observed in 7 (26%) and 3 (11%) respectively. Among an age- and stage-matched cohort of 28 TNBC patients without NY-ESO-1 expression, the median CD8 count was 98.7cells/mm^2^ (range 0 to 828.7 cells/mm^2^). 10 of these 28 patients (36%) had a brisk CD8 infiltrate, while a moderate or minimal infiltrate was observed in 12 (43%) and 6 (21%) respectively. Figure 5 shows the relationship between NY-ESO-1 expression and degree of CD8 tumor infiltrating lymphocytes in TNBC.

The NY-ESO-1 positive patients tended to have higher CD8 counts (mean/SD: 428.1 cells/mm^2^/419) versus the negative patients (mean/SD: 195.7 cells/mm^2^/217) [p = 0.018]. Although, it did not reach statistically significance, there was a positive correlation between NY-ESO-1 antibody density and CD8 count in the 8 patients whose sera was positive for NY-ESO-1 antibodies (p = 0.068).

### NY-ESO-1 Induction and Hypomethylation *in vitro*

As CT antigens are regulated by DNA methylation,[24] we determined the ability to induce NY-ESO-1 expression *in vitro* by treating MCF-7 breast cancer cell lines with hypomethylating agents (decitabine and 5-azacytidine) at 0 and 48 hours. DNA and RNA were extracted for analyses at 72 hours. Figure 6 shows the effective induction of NY-ESO-1 by both agents. In addition, quantitative pyrosequencing of NY-ESO-1 promoter showed that the percentage of NY-ESO-1 promoter methylation was reduced by both decitabine and 5-azacytidine in MCF-7 cells (Figure 7).

## Discussion

Therapeutic options for individuals with TNBC remain limited. In addition, the clinical outcome of these patients is poorer than the outcome of those with hormone receptor positive breast cancer. Due to that fact that tumors express specific antigens that can be recognized by T-cells,[25], [26] anti-cancer vaccines may have a role to play in management of these patients if an appropriate target antigen and patient population can be identified. Due to its high degree of immunogenicity, NY-ESO-1 has emerged as one of the important targets for immunotherapy. Although, a number of studies have sought to determine the frequency of NY-ESO-1 expression in primary breast cancers, several of them have been flawed as they have not selected patients based on hormone or HER2 receptor status.[5], [9], [20], [27], [28], [29], [30], [31] The current study represents the largest and most rigorous investigation on NY-ESO-1 CT antigen in patients with TNBC. In addition, while a number of clinical trials utilizing various forms of vaccines against NY-ESO-1 have been completed, no specific breast cancer trial using this strategy has been implemented. Our results show that NY-ESO-1 CT antigen can be detected by IHC in up to 16% of TNBC patients, as opposed to only 2% of patients with hormone receptor positive disease and thus is a valid target for immunotherapy in the TNBC sub-group of patients.

A limitation of our study was the inclusion of 66% of the TNBC cohort having earlier stages (1/2) of cancer vs. the comparison ER+ group without any stage 1 or 2 patients. This may have resulted in a bias in the results as, theoretically, early stage of disease may have higher rates of NY-ESO-1 expression or other immune responses before host immune tolerance to tumor is established. When tumor grows or disease progresses, host immune tolerance to tumor antigens start to be established due to immunosuppressive mechanism such as induction of immunosuppressive cell populations and cytokines, and immune checkpoints. Thus, expression of tumor antigen or immune response may potentially be down regulated during a later stage of disease.

Our data did not suggest any relationship between clinical outcome and NY-ESO-1 antigen expression, or antibody response. While this may be due to the low event rate in our cohort, it is also possible that CT antigen expression has a neutral effect on breast cancer prognosis as also reported for ovarian cancer.^19^ Nevertheless, the NY-ESO-1 positive patients tended to have higher tumor infiltration by CD8+ T cells, and those with NY-ESO-1 antibody trended towards increased density of CD8+ T cells, indicating a potential link between NY-ESO-1 immunity and infiltration of TNBC by T cells. This is an important consideration as the presence of intratumoral T cells has been shown to correlate with improved survival in patients with several cancer types such as ovarian, colon, lung, melanoma, and breast cancers.[32], [33], [34], [35], [36], [37] Moreover, tumor infiltrating lymphocytes have been shown to predict response to anthracycline-based chemotherapy in estrogen receptor-negative breast cancer.[38].

We also showed that induction of expression of NY-ESO-1 *in vitro* using hypomethylating agents is feasible. This could be of considerable clinical significance as it may be reasonable to hypothesize that induction of NY-ESO-1 *in vivo* utilizing a similar strategy is possible. In fact, a recently published phase I trial of decitabine with carboplatin in ovary cancer patients showed DNA hypomethylating activity with a decitabine-based regimen.[39] Several other clinical trials employing a similar strategy of hypomethylation or other epigenetic modulation to re-express ‘silenced’ hormone receptors or tumor suppressor genes in breast carcinomas are underway. The question as to whether inducing NY-ESO-1 *in vivo* will augment spontaneous or vaccine induced immunity to NY-ESO-1 requires further investigation.

The high frequency of 73% of NY-ESO-1 antibodies in NY-ESO-1 expressing TNBC patients in this present study was unexpected. Although surprising, these findings are however accurate given the similar high antibody frequency also observed in the close homolog, LAGE-1. Other studies showed that antibodies to NY-ESO-1 were demonstrable in 0.9% to 4% of unselected primary breast cancer patients’ sera.[7], [8], [27] Hamaï et al. showed that 12 of 1374 (0.9%) primary unselected breast cancer patients had detectable antibodies to NY-ESO-1. Of those who had tissue available, 7 of 8 (88%) were found to be NY-ESO-1 positive by PCR, and had high-grade ER negative disease. The patient whose breast cancer was NY-ESO-1 negative was diagnosed with melanoma very soon afterwards. This suggests that the development of an immune response to NY-ESO-1 is specific to those whose tumors are positive for the antigen and may be found in a higher proportion of properly selected patients. Other studies in ovary cancer have shown antibody rates of up to 12.5% to 30% in patients with NY-ESO-1 positive tumors.[19], [40] This study by Stockert et al. showed a frequency of up to 50% in patients with more advanced NY-ESO-1 expressing tumors.

The implications of our findings are twofold. First, these results suggest that the subset of patients with TNBC whose tumors express NY-ESO-1 have particularly high inherent immunogenicity and therefore are an attractive population for CT vaccine studies. A spontaneous antibody response to NY-ESO-1 antigen has been shown to predict both CD8 and CD4 T cell response to NY-ESO-1.[17], [41] This will imply a possible clinical benefit given that CD8 T cells are critical in tumor rejection.[42] The other ramification of these results is the possibility of utilizing an NY-ESO-1 vaccine strategy in patients with TNBC to boost any preexisting immunity to NY-ESO-1. This will likely assist with T cell expansion/activation and the potential for co-stimulatory blockade of negative immune regulatory factors and subsequently the induction of improved host tumor rejection. In addition to T cell receptor recognition, the optimal activation of a T cell involves signals provided by co-stimulatory molecules which are balanced by negative co-stimulatory molecules. It is clear that several factors including cytotoxic lymphocyte antigen–4:B7 (CTLA-4:B7),[43] programmed death-1 (PD-1),[44], [45] and lymphocyte activation gene-3 (LAG 3) all negatively regulate T cell activation. For example, PD-LI, the ligand for PD-1 is expressed in up to 50% of invasive ductal carcinomas and is associated with worse prognostic indices.[46] Grosso et al. showed that LAG 3 blockade resulted in increased accumulation and function of antigen-specific CD8 T cells within organs and tumors that express their cognate antigen. In addition, combining LAG 3 blockade with specific antitumor vaccines resulted in an increase in activated CD8 T cells in the tumor and disruption of the tumor parenchyma.[47] Similarly, ipilimumab, a monoclonal antibody against CTLA-4 also utilized as a therapy for patients with malignant melanoma has been shown to result in the most clinical benefit in patients with pre-existing NY-ESO-1 immunity.[48] Together, targeting negative T cell co-stimulatory pathways with immunotherapy directed against NY-ESO-1 in patients with TNBC may be a novel way to ultimately improve clinical outcomes for this subset of patients.

In conclusion, our study reveals that NY-ESO-1 is expressed in a subset of TNBC patients and leads to a relatively high spontaneous humoral immune response rate in these individuals. Although the influence of NY-ESO-1 positivity on the OS or PFS of TNBC patients is likely neutral as evidenced by this present study, the potential for augmenting an individual’s immunity by interfering with negative T cell co-stimulatory pathways is worthy of further investigation. Given these observations, we propose that patients with TNBC may benefit from targeted therapies directed against NY-ESO-1.
