# Correction: NY-ESO-1 Cancer Testis Antigen Demonstrates High Immunogenicity in Triple Negative Breast Cancer

**DOI:** 10.1371/annotation/5cdf6105-2a52-497a-86b3-db8f4a4e439c

**Published:** 2012-08-09

**Authors:** Foluso O. Ademuyiwa, Wiam Bshara, Kristopher Attwood, Carl Morrison, Stephen B. Edge, Christine B. Ambrosone, Tracey L. O’Connor, Ellis G. Levine, Anthony Miliotto, Erika Ritter, Gerd Ritter, Sacha Gnjatic, Kunle Odunsi

Two authors, Adam R. Karpf, and Smith A. James, were inadvertently omitted from the by-line and Author Contributions section. The correct author byline is: Foluso O. Ademuyiwa 1*, Wiam Bshara 2, Kristopher Attwood 3, Carl Morrison 2, Stephen B. Edge 4, Adam R. Karpf 5, Smith A. James 5, Christine B. Ambrosone 6, Tracey L. O’Connor 1, Ellis G. Levine 1, Anthony Miliotto 7, Erika Ritter 8, Gerd Ritter 8, Sacha Gnjatic 8, Kunle Odunsi 7.

The correct affiliation list is: 1 Department of Medicine, Roswell Park Cancer Institute, Buffalo, New York, United States of America, 2 Department of Pathology, Roswell Park Cancer Institute, Buffalo, New York, United States of America, 3 Department of Biostatistics, Roswell Park Cancer Institute, Buffalo, New York, United States of America, 4 Department of Surgical Oncology, Roswell Park Cancer Institute, Buffalo, New York, United States of America, 5 Department of Pharmacology & Therapeutics, Roswell Park Cancer Institute, Buffalo, New York, United States of America 6 Department of Cancer Prevention and Control, Roswell Park Cancer Institute, Buffalo, New York, United States of America, 7 Department of Gynecologic Oncology, Roswell Park Cancer Institute, Buffalo, New York, United States of America, 8 Ludwig Institute for Cancer Research Ltd. New York Branch of Human Cancer Immunology, Memorial Sloan Kettering Cancer Center, New York, New York, United States of America

Author contributions are: ARK Conceived and designed the experiments, performed the experiments, analyzed the data, contributed reagents/materials/analysis tools, and wrote the paper. SAJ performed the experiments, analyzed the data, and wrote the paper 

